# Formation and Chemical Structure of Carbon-13 Tracer Lignin-Carbohydrate Complexes (LCCs) During Kraft Pulping

**DOI:** 10.3390/molecules30051077

**Published:** 2025-02-26

**Authors:** Zhi Wang, Yimin Xie, Boxuan Zhao

**Affiliations:** 1 Research Institute of Pulp & Paper Engineering, Hubei University of Technology, Wuhan 430068, China; ppzwang2022@163.com (Z.W.); ppbxzhao@163.com (B.Z.); 2 Hubei Provincial Key Laboratory of Green Materials for Light Industry, Hubei University of Technology, Wuhan 430068, China

**Keywords:** carbon-13 labeling, lignin model compound, kraft pulping, xylose, LCC

## Abstract

In this study, a modified synthetic method for labeling a lignin dimer (guaiacylglycerol-β-guaiacyl ether-[α-^13^C]) was developed. The chemical structure of the target compound was analyzed using ^1^H-NMR, ^13^C-NMR, and other analytical techniques. Then, the ^13^C-labeled phenolic lignin model compound was subjected to kraft pulping in the presence of xylose. Finally, the resulting reaction products were fractionated using acid precipitation and ethyl acetate extraction, and each fraction was analyzed by carbon-13 nuclear magnetic resonance (^13^C-NMR) and two-dimensional heteronuclear multiple quantum coherence (HMQC) spectroscopy. This aimed to investigate the occurrence of lignin–carbohydrate complexes (LCCs) during the conventional kraft pulping process. Employing ethanol as the reaction medium facilitated the bromination of 4-acetylguaiacol-[α-^13^C], resulting in a homogeneous reaction and significantly improving the yield of the brominated product to over 90%. Additionally, kraft pulping of the phenolic lignin model compound in the presence of xylose led to the occurrence of minor quantities of benzyl ether-type lignin–carbohydrate complex (LCC) structures, which were predominantly detected in the ethyl acetate extractive.

## 1. Introduction

A significant challenge in lignin structure research is the difficulty in sample separation. Due to the complex macromolecular structure of lignin, which is closely associated with cellulose and hemicellulose, it is highly susceptible to degradation and condensation during the separation process. Consequently, isolating protolignin remains nearly impossible [1]. Lignin and lignin–carbohydrate complexes (LCCs) can be extracted using the Björkman method [2,3]. However, owing to their complex structure, it is difficult to understand the occurrence and cleavage of chemical bonds during the kraft pulping process. Therefore, it is important to apply low-molecular-weight lignin model compounds to investigate chemical structural changes at the molecular level. The most important bonding type in lignin molecules is the arylglycerol-β-aryl ether (β-O-4) structure, which accounts for approximately half of the bonds between structural units in softwood lignin [4]. Therefore, studying its degradation mechanism under different conditions is helpful to develop more efficient lignin degradation technology and realize the full utilization of biomass resources, which is also important in the pulp and paper industry [5].

cLCCs are an important component of residual lignin in kraft pulp, making it resistant to further degradation and posing a significant obstacle to improving pulp whiteness [6,7]. Some LCCs originate from the fiber material itself, while others result from condensation reactions between lignin and carbohydrates during the kraft pulping process. Therefore, it is necessary to investigate the mechanisms of occurrence of LCC in kraft pulping and analyze its structural changes [8,9,10]. This research is crucial for developing environmentally friendly pulping technology [11,12].

The use of lignin model compounds is a well-established method for investigating changes in lignin during pulping, bleaching, and other processing stages. In previous research, we studied the behavior of condensed lignin model compounds during the kraft cooking process and discovered the formation of a new LCC structure involving benzyl ether bonds in the presence of xylose [13]. Furthermore, it is necessary to investigate the possibility of condensation in an uncondensed β-O-4 lignin model with carbohydrates during kraft cooking.

Based on previous research [14], this study explored a modified synthesis method for the β-O-4 lignin model compound with ^13^C isotope labeling, specifically a 4-(α-bromoacetyl)-guaiacol-[α-^13^C] intermediate. Meanwhile, the purification methods of the intermediate product 4-(α-bromoacetyl)-guaiacol-[α-^13^C] were improved. The reaction in this step was transformed from heterogeneous to homogeneous. Therefore, the efficiency of the synthesis of the β-O-4 lignin model compounds was also improved. This made it possible to trace the changes in the α-carbon atom of the lignin side chain. The synthesized guaiacylglycerol-β-guaiacyl ether-[α-^13^C] was subjected to kraft pulping in the presence of xylose, and the formation of complexes between the lignin model compound and xylose was analyzed. Furthermore, the bonding type between the α position of the lignin side chain and xylose was investigated in detail.

## 2. Results and Discussion

### 2.1. Modification of the Synthesis Method of Guaiacylglycerol-β-Guaiacyl Ether-[α-^13^C]

#### 2.1.1. Improvement in Bromination Reaction Solvent

During the synthesis of intermediate 4-(α-bromoacetyl)-guaiacol-[α-^13^C], previous studies by Hu [15] used elemental bromine as the brominating agent. However, controlling the dosage of bromine addition was challenging, often resulting in incomplete bromination or excessive bromine, which led to hydrogen substitution on the benzene ring. Research conducted by Caroll and Kenneth [16,17] on selective bromination reactions demonstrated that using copper bromide in a chloroform/ethyl acetate solvent mixture provided excellent selectivity for the bromination of the side chains in 4-hydroxybenzophenone derivatives. This reaction was performed without nitrogen protection. Hu [15] further confirmed that using copper bromide as a brominating agent stabilized the bromination rate of 4-acetylguaiacol at 70–80%. This modification increased the yield of 4-(α-(2-methoxyphenoxy)-acetyl)-guaiacol to nearly twice that of the conventional method.

In this study, ethanol was used as the reaction solvent for the synthesis of 4-(α-bromoacetyl)-guaiacol-[α-^13^C]. The bromination efficiency and yield of guaiacylglycerol-β-guaiacyl ether were significantly improved. Previous studies [18] have demonstrated the excellent solubility of copper bromide in ethanol. The improvement in bromination can be attributed to the superior solubility of Cu(II) bromide in ethanol. Additionally, the hydrobromic acid produced during the reaction remained dissolved in the solvent. The absence of observable hydrobromic acid release during the reaction confirmed that most of the produced acid remained dissolved in the reaction medium. This change in solvent system from chloroform/ethyl acetate to ethanol effectively transformed the bromination process from a heterogeneous to a homogeneous reaction. As a result, the yield of the target compound consistently exceeded 94%, representing an improvement of 14% compared to the previous work using a chloroform/ethyl acetate mixed-solvent system.

#### 2.1.2. Optimization of Bromination Reaction Time

As shown in Table 1, during the first hour, the bromination reaction proceeded rapidly, and extending the reaction time beyond this period had minimal impact on the bromination rate. When ethanol was used as the reaction solvent, the bromination reaction was completed within 1 h, and prolonging the reaction time did not significantly affect the yield of the product. However, the amount of recovered cuprous bromide does not accurately reflect the reaction process. Notably, the lowest recovery rate of cuprous bromide (72.7%) coincided with the highest product yield, indicating the presence of excess copper bromide and hydrogen bromide in the reaction mixture. The findings suggest that as long as hydrogen bromide gas does not escape from the reaction system, a higher product yield can be achieved.

#### 2.1.3. Purification of Bromination Product

Previous studies [19,20] have suggested that drying the filtrate obtained after the reaction, followed by recrystallization with benzene, could effectively purify the bromination product. However, as shown in Table 1, cuprous bromide could not be fully recovered through filtration, with a portion of it remaining in the filtrate. This may explain why the product could not be obtained via recrystallization from benzene. In this study, silica gel chromatography was used to separate the brominated products. The results showed that the brominated product could be successfully purified as crystals when ethyl acetate/n-hexane (1/2, *v*/*v*) was used as the eluent.

#### 2.1.4. Analysis of 4-(α-Bromoacetyl)-Guaiacol-[α-^13^C]

**Figure 1:**
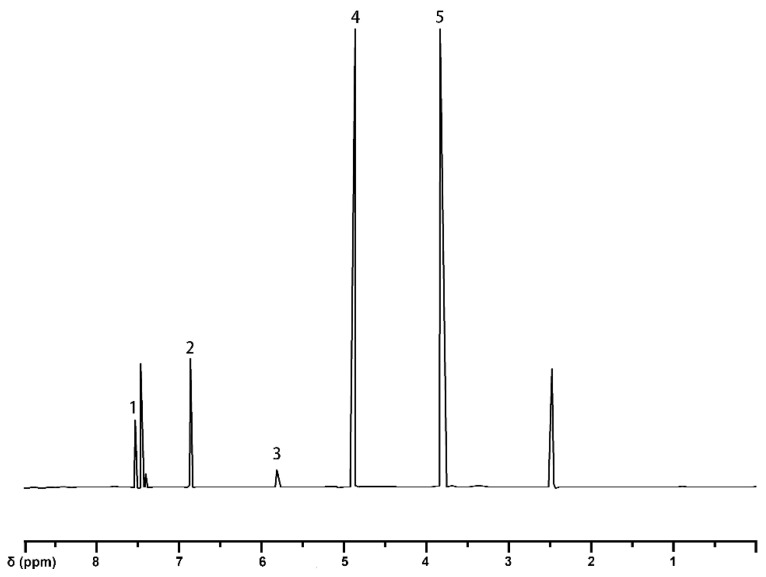
^1^H-NMR spectrum of 4-(α-bromoacetyl)-guaiacol.

According to Vanucci et al. [21], the ^1^H-NMR spectrum of the compound 4-(α-bromoacetyl)-guaiacol-[α-^13^C] (III) (Figure 1) shows two distinct signals for the methoxy (-OCH_3_) groups at δ3.82 ppm and δ3.83 ppm, respectively. A characteristic signal at δ5.26 ppm confirms the presence of a -CH_2_ structure, while signals in the range of δ6.85–δ7.57 ppm, with overlapping peaks, indicate the presence of aromatic protons (Table 2). Based on this, the product was determined to be 4-(α-bromoacetyl)-guaiacol-[α-^13^C] (III). This also indicates that the synthesis of compound III can be successfully achieved by replacing the trichloromethane/ethyl acetate mixture with ethanol as the reaction solvent.

#### 2.1.5. Analysis of Guaiacylglycerol-β-Guaiacyl Ether-[α-^13^C]

**Figure 2:**
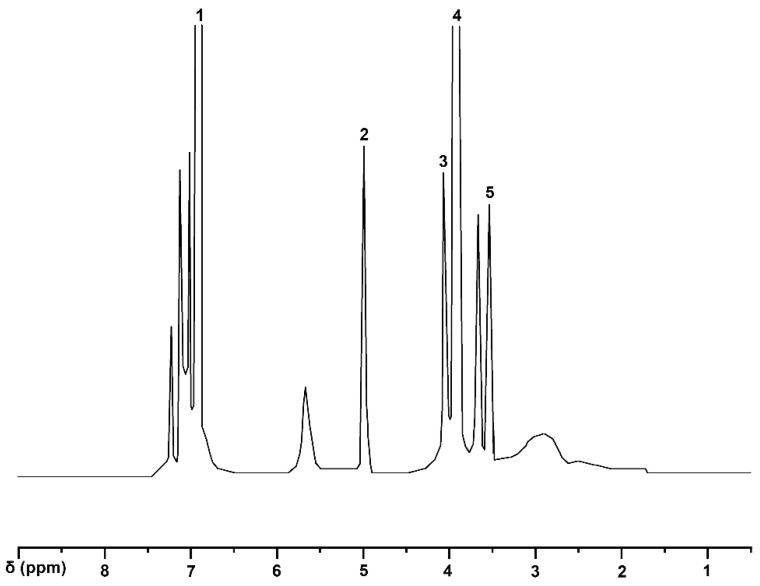
^1^H-NMR spectrum of guaiacyl glycerol-β-guaiacyl ether.

The ^1^H-NMR spectra of guaiacylglycerol-β-guaiacyl ether-[α-^13^C] (VI) (Figure 2) were analyzed based on the report by Castellan et al. [22], and the signals of two methoxy groups (-OCH_3_) were observed at δ3.70–δ3.76 ppm. A peak at δ4.73 ppm indicates the presence of a -CH group at the α position. The signals in the δ6.68–δ7.68 ppm range exhibit overlapping peaks, corresponding to aromatic protons, and a total of seven aromatic protons were identified. According to Katahira and Sipila et al. [23,24], the two peaks observed at δ4.25–δ4.30 ppm correspond to β-position protons, with one proton. The signals at δ3.23–δ3.61 ppm correspond to γ-position protons with two protons. The specific information is shown in Table 3.

**Figure 3:**
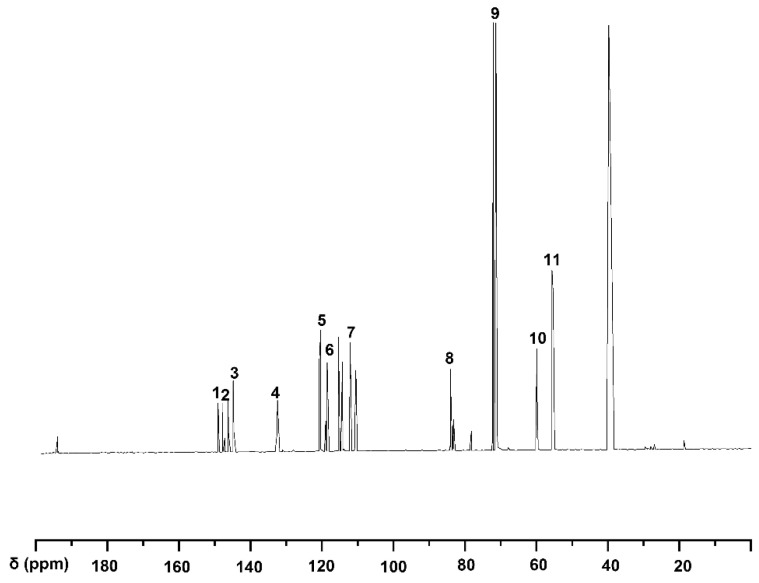
^13^C-NMR spectrum of guaiacyl glycerol-β-guaiacyl ether-[α-^13^C] (VI).

The ^13^C-NMR spectrum of compound VI is shown in Figure 3. According to reference [25], characteristic signals at δ149.83–δ149.87 ppm, δ148.23–δ148.53 ppm, δ145.561–δ147.123 ppm, δ133.13–δ133.42 ppm, δ120.80–δ119.64 ppm, and δ111.10–δ115.89 ppm correspond to C_3_, C_3_/C_4_, C_3_/C_5_, C_1_, C_6_, C_5_, and C_2_, respectively. A distinct signal at δ83.80–δ84.61 ppm was assigned to C_β_(β-O-4), while signals in the range of δ70.27–δ71.74 ppm were assigned to C_α_(β-O-4), showing a strong resonance due to the ^13^C labeling at the C_α_ position. Additionally, a signal at δ60.22 ppm corresponds to C_γ_(β-O-4), and the OCH_3_ groups were confirmed by the signal at δ55.52–δ55.72 ppm (Table 4). Based on the combined ^1^H-NMR and ^13^C-NMR analyses, the product VI synthesized through the modified method is guaiacylglycerol-β-guaiacyl ether-[α-^13^C] with high purity.

### 2.2. Co-Polymerized Products of Guaiacylglycerol-β-Guaiacyl Ether-[α-^13^C] with Xylose in Kraft Pulping Process

**Figure 4:**
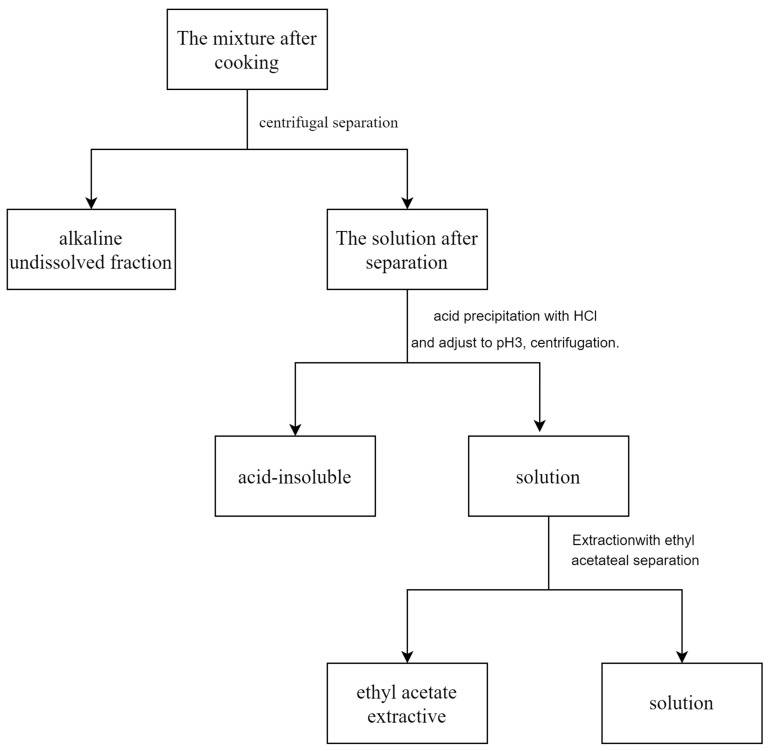
Classification of cooking products from lignin model compound in presence of xylose.

Alkali-insoluble substances cannot be obtained from the cooking process, indicating that both β-O-4-type lignin model compounds and xylose derivatives are dissolved in the black liquor. After acid precipitation and centrifugation, acid-insoluble substances were obtained. The classification process is shown in Figure 4. Analysis with ^13^C-NMR showed that the acid-soluble fraction contains a large amount of undegraded xylose. Furthermore, the acid-soluble fraction is rich in undegraded xylose components. According to certain studies [26], under conditions when xylose is present, the benzyl ether-type LCC structure formed during the cooking of the condensed-type lignin model compounds using the kraft method mainly exists in the ethyl acetate extractive. Consequently, the analysis of the products primarily investigates the ethyl acetate extractive.

**Figure 5:**
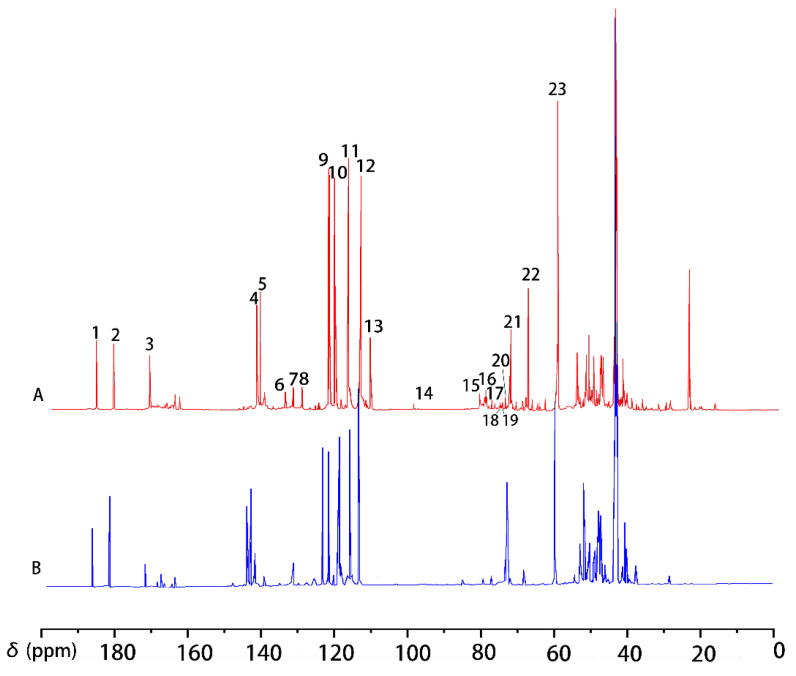
^13^C-NMR spectra of ethyl acetate extractives from guaiacyl glycerol-β-guaiacyl ether-[α-^13^C] by kraft cooking ((**A**): adding xylose (red); (**B**): control experiment(blue)).

The ^13^C-NMR spectrum of ethyl acetate extractive (Figure 5A) shows that the signal at δ66.0 ppm (No.22) corresponds to xylose C5. Signals at δ98.7 ppm (No.14), δ77.8 ppm (No.17), δ76.5 ppm (No.18), δ75.6–74.2 ppm (No.19), and δ72.8 ppm (No.20) correspond to C1, C3/C4, C3, C2, and C2/C3, respectively, in xylose. The intensity of signal No.22 was relatively high, indicating that the ethyl acetate extractive contained xylose components. The signal at δ109.7–δ147.8 ppm in Figure 5 corresponds to aromatic substances, indicating that the ethyl acetate extractive also contains lignin derivatives. The ^13^C-NMR spectrum (Figure 5B) of the ethyl acetate extractive from guaiacylglycerol-β-guaiacyl ether-[α-^13^C] after kraft cooking (control experiment) showed that the intensity of peak No.3 in Figure 5A (170.6 ppm) was stronger than that of peak No.3 in Figure 5B (171.5 ppm) [27,28,29]. This indicates that a new carbonyl signal from xylose derivatives, such as uronic acid, was attacked by HS- and OH- during kraft cooking. In addition, new signals at δ81.5 ppm (No.15) and δ79.3 ppm (No.16) appear in Figure 5A, which are derived from C_α_ and C_β_ on the new benzyl ether-type LCC linkage [30], indicating the existence of a newly formed LCC structure in the ethyl acetate extractive. Table 5 shows the above signals.

**Figure 6:**
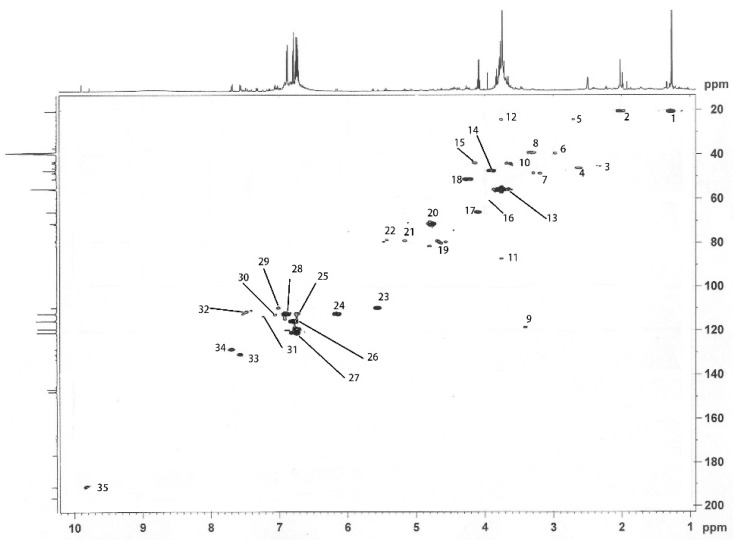
Two-dimensional HMQC spectrum of ethyl acetate extractive from guaiacyl glycerol-β-guaiacyl ether-[α-^13^C] and xylose by kraft cooking.

The 2D HMQC spectrum of the ethyl acetate extractive from the cooking process, as shown in Figure 6, indicates that peak No.21 (δC/δH 79.3/5.15) and peak No.22 (δC/δH 79.3/5.40) correspond to C_α_-H in the ferulic acid-linked LCC structure [31,32]. Peaks No.8 (δC/δH 39.5/3.2–3.38), No.16 (δC/δH 60.0/3.97), No.17 (δC/δH 66.0/4.1), No.19 (δC/δH 80.1/4.65), and No.20 (δC/δH 71.3/4.78) correspond to CH connected to the aromatic ring, C_γ_-H or C_5_-H of guaiacyl structures or uronic acid, C_γ_-H or C_5_-H of guaiacyl structures or uronic acid, C_α_-H or C_2_-H of guaiacyl structures or uronic acid, and C_α_-H or C_3_-H/C_4_-H of guaiacyl structures or uronic acid, indicating that xylose derivatives such as uronic acids are generated during the kraft pulping process. Thus, the ethyl acetate extractive from the kraft pulping of guaiacyl glycerol-β-guaiacyl ether-[α-^13^C] and xylose contains newly formed benzyl ether-type LCC structures. This is consistent with the inference about Figure 5.

### 2.3. Formation Mechanism of LCC Structure During the Kraft Cooking Process

**Figure 7:**
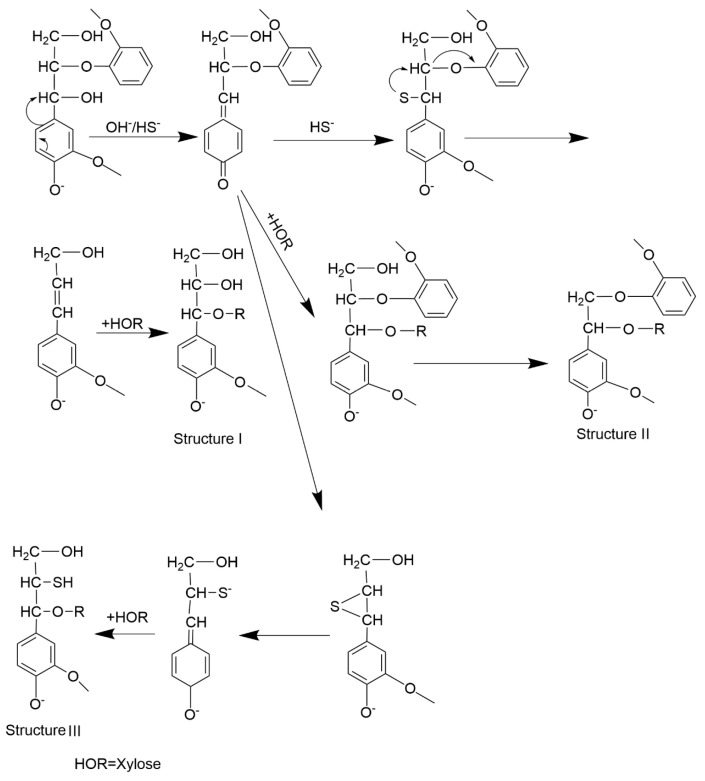
LCC structure formation pathway of LCC structure between phenolic lignin model and xylose (HOR) during kraft cooking.

Based on the reaction mechanism of kraft cooking, the LCC structure is formed between the phenolic lignin model and xylose during cooking (Figure 7). The process of its formation is summarized as follows: The xylose ring opens during kraft cooking to form xylose derivatives, mainly uronic acid. This is because xylose derivatives are rich in hydroxyl groups. In the process of kraft cooking, it can easily be dehydrogenated to form a quinomethide intermediate and further produces LCC structures by attacking hydroxyl groups of xylose derivatives [33,34]. Therefore, structure I, structure II, and structure III may be generated after a part of C-γ is eliminated. However, only a weak signal of the newly formed benzyl ether-type LCC structure is observed by the ^13^C tracer method, indicating that the content of the newly formed LCC structure is not rich in the ethyl acetate extractive.

## 3. Experiment

### 3.1. Materials

The experimental instruments used in this study included an X-6 micro melting point tester (Beijing TECK Instrument Co., Ltd., No. 11 Tao Yang Road, Yongdingmen, Dongcheng District, Beijing, China); EYELAN-1000 vacuum rotary evaporator (Shanghai Ailang Instrument Co., Ltd., No. 1630 Yecheng Road, Jiading Industrial Zone, Shanghai, China); and an NMR Bruker Avance III HD 600 spectrometer (40 Manning Road, Billerica, MA, USA).

The experimental reagents used in this study, including phosphoric acid (85%), diphosphorus pentoxide, anhydrous sodium sulfate, anhydrous ether, anhydrous ethanol, trichloromethane, ethyl acetate, anhydrous calcium chloride, metallic sodium, anhydrous potassium carbonate, formaldehyde, dilute hydrochloric acid, benzene, and sodium hydroxide, were purchased from Tianjin Damao Chemical Reagent Factory (Huaming Street, Dongli District, Tianjin, North Yubao Village West (No.5 East Industrial Zone), Tianjin, China). Anhydrous sodium acetate (1-^13^C, 99%) was purchased from Beijing Shubowei Chemical Instrument Co., Ltd. (Room 204D, 9 Beiwei Road, Xuanwu District, Beijing, China). Guaiacol, copper bromide, and sodium borohydride were purchased from China Pharmaceutical Group Shanghai Chemical Reagent Company (52 Ningbo Road, Huangpu District, Shanghai, China)).

### 3.2. Methods

#### 3.2.1. Synthesis of lignin dimers-[α-^13^C]

**Figure 8:**
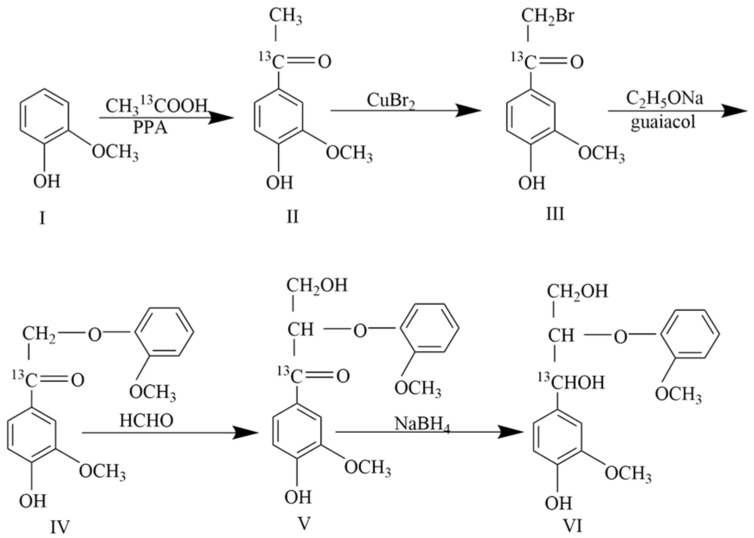
Synthesis route of guaiacyl glycerol-β-guaiacyl ether-[α-^13^C].

The synthesis of guaiacyl glycerol-β-guaiacyl ether-[α-^13^C] was primarily based on the methods established by Nakatsubo and Nakano [35,36], with several key steps modified. The synthetic route is shown in Figure 8.

#### 3.2.2. Synthesis of 4-Acetyl Guaiacol-[α-^13^C]

A total of 90 g of phosphoric acid (85%) was poured into a round-bottom flask containing anhydrous phosphorus pentoxide (85%) and heated with stirring in an oil bath at 100 °C for 2h, forming polyphosphoric acid (PPA). Subsequently, 5 g of isotope-labeled sodium acetate (1-^13^C, 99%) was thoroughly mixed with an excess of guaiacol (12.5 g). Then, the hot PPA was quickly poured into the mixture and vigorously stirred at 100 °C for 15 min. After completion, the mixture was cooled with ice water, extracted with diethyl ether until the extractive became colorless, and then dried with anhydrous sodium sulfate. Following the removal of ether by rotary evaporation under reduced pressure and subsequent recrystallization from ethanol, the resulting crystals were vacuum-dried to obtain pale yellow crystals of 4-acetylguaiacol-[α-^13^C]. The melting point of the product was determined to be in the range of 114.2–115.5 °C, yielding 44.7%.

#### 3.2.3. Synthesis of 4-(α-Bromoacetyl)-Guaiacol-[α-^13^C]

At 50 °C, copper bromide (4.467 g) was dissolved in 50 mL of ethanol (or a chloroform/ethyl acetate (1/1, *v*/*v*) mixed solvent) and added dropwise to a mixture of 4-acetyl guaiacol-[α-^13^C] (1.992 g) dissolved in 50 mL of ethanol (or the chloroform/ethyl acetate (1/1, *v*/*v*) mixed solvent). White cuprous bromide was produced, and the reaction process was monitored using thin-layer chromatography (TLC). Upon completion of the reaction, the reaction mixture was filtered, and the filtrate was evaporated under vacuum to remove ethanol (or the chloroform/ethyl acetate (1/1, *v*/*v*) mixed solvent). The filtrate was then extracted with chloroform and dried with anhydrous sodium sulfate. The chloroform filtrate was evaporated under vacuum, and the obtained crude product was purified using column chromatography. Light yellow crystals were obtained using n-hexane/ethyl acetate (2/1, *v*/*v*) as the eluent. When ethanol was used as the solvent, the yield of 4-(α-bromoacetyl)-guaiacol-[α-^13^C] was 94.8% and the melting point was 69.9–74.4 °C. When chloroform/ethyl acetate (1/1, *v*/*v*) was mixed with the solvent, the yield was 81.54% and the melting point was 73.6–76.6 °C.

#### 3.2.4. Synthesis of 4-(α-(2-Methoxyphenoxy)-Acetyl)Guaiacol-[α-^13^C]

Under continuous stirring, 7.0 g of sodium metal was quickly added to a round-bottom flask containing 250 mL of anhydrous ethanol. To prevent the volatilization of anhydrous ethanol during the reaction, the flask was equipped with a condenser. While stirring at 55 °C, 40 g of guaiacol was slowly added dropwise to the freshly prepared sodium ethylate solution. After the addition was complete, the solution was evaporated under vacuum to obtain sodium guaiacolate. The sodium guaiacolate was completely dissolved in 175 mL of anhydrous dimethylformamide (DMF). Then, 5 g of 4-(α-bromoacetyl)-guaiacol-[α-13C] was dissolved in 35 mL of anhydrous DMF and mixed with the prepared sodium guaiacolate solution. After a few minutes of reaction, the solution was poured into ice water, while the pH value of the solution was adjusted to 3.0 with dilute hydrochloric acid. Then, the solution was extracted several times with chloroform. The chloroform solution was dehydrated with anhydrous sodium sulfate and then evaporated under high vacuum to remove the DMF by addition of xylene to form azeotrope. The residue was separated using silica gel column chromatography, initially eluting with n-hexane/ethyl acetate (4/1, *v*/*v*), followed by benzene/ethyl acetate (3/1, *v*/*v*) after the main impurities were removed. The separated fraction was evaporated under vacuum, and a few drops of ether were added to induce crystallization. The resulting 4-(α-(2-methoxyphenoxy)-acetyl)-guaiacol-[α-13C] appeared as light yellow crystals with a yield of 82.80%. The melting point range was 87.3–91.9 °C.

#### 3.2.5. Synthesis of 4-(α-(2-Methoxyphenoxy)-β-Hydroxypropionyl)-Guaiacol-[α-^13^C]

To a solution of 3 g of 4-(α-2-methoxyphenoxy)-acetyl)-guaiacol-[α-^13^C] in 30 mL of ethanol, 1.7 g of anhydrous potassium carbonate and 30 mL of formaldehyde were added. The mixture was stirred at 50 °C for 2 h. After the reaction, distilled water was added to the solution, and the pH was adjusted to 3.0 using dilute hydrochloric acid. The solution was then extracted with chloroform and dried using an anhydrous sodium sulfate. The chloroform solution was evaporated under vacuum. The residue was purified by silica gel column chromatography using a mixture of benzene and ethyl acetate (3/1, *v*/*v*) as the effluent. The obtained fractions were evaporated under vacuum and a few drops of ether were added to crystallize the product 4-(α-(2-methoxyphenoxy)-β-hydroxypropioyl)-guaiacol-[α-^13^C]. The product was then filtered and vacuum-dried. The product yield was 67.2% and the melting point range was 98.2–101.1 °C.

#### 3.2.6. Synthesis of Guaiacylglycerol-β-Guaiacyl Ether-[α-^13^C]

Under stirring, 1 g of 4-(α-(2-methoxyphenoxy)-β-hydroxypropioyl)-guaiacol-[α-^13^C] was dissolved in 100 mL of 0.1 mol/L NaOH solution in a nitrogen atmosphere. Then, 0.17 g of sodium borohydride was added, and the reaction was carried out at room temperature overnight. After the reaction, the solution was neutralized with dilute hydrochloric acid to a pH of 7.0 and then extracted with chloroform. The chloroform layer was washed several times with distilled water and dried using anhydrous sodium sulfate. After evaporation under vacuum, a syrupy residue was obtained. The dimer of lignin (guaiacylglycerol-β-guaiacyl ether-[α-^13^C]) was obtained after crystallization with ethyl ether. The yield of the product was 65.16%. The melting point of the product was 99.7–102.9 °C. Since the melting point of the threo-type product is 118–120 °C and that of the erythro-type product is 93–94 °C, the product obtained in this study was a mixture of both the threo- and erythro-types. The total yield of the product was 15.38%, and the ratio of the α-^13^C isotope was 99%.

#### 3.2.7. The Cooking Experiment of Lignin Dimer and Xylose

A total of 400 mg of dimer of lignin and 400 mg of xylose were placed into a 12 mL stainless steel autoclave. The mixture was heated in an oil bath with 17% active alkali (as Na2O), 25% sulfidity, and a 1:4 wood-to-water ratio. The temperature was gradually increased to 160 °C at a rate of 10 °C per 5 min and maintained at 160 °C for 1 h. After the cooking process, the reactor was cooled with water, and the cooked products were graded using acid precipitation. The reaction mixture was classified into four fractions: alkali-insoluble, acid-insoluble, acid-soluble, and ethyl acetate extractives. These fractions were analyzed using ^13^C-NMR and two-dimensional HMQC.

#### 3.2.8. ^13^C-NMR and Two-Dimensional HMQC Analysis

The prepared samples were dissolved in DMSO-d6, and their ^13^C-NMR, 1H-NMR, and two-dimensional HMQC spectra were determined using a Bruker Avance-600 nuclear magnetic resonance spectrometer equipped with CryoProbesTM and a φ5 mm sample tube. The measurement parameters were set as follows: For ^13^C-NMR, the frequency was 150 MHz with an acquisition time (AQ) of 0.94 s, pulse delay (PD) of 1.75 s, and 2000 number of scans (NS). For ^1^H-NMR, the frequency was 600 MHz with an AQ of 0.3 s, PD of 4.0 s, and 100 NS.

## 4. Conclusions

(1) Guaiacyl glycerol-β-guaiacyl ether-[α-^13^C] was successfully synthesized using sodium acetate-1-^13^C and guaiacol as raw materials. When copper bromide was employed as the brominating agent and ethanol was the solvent, the side chain of the intermediate product, 4-acetyl guaiacol-[α-^13^C], was brominated with high selectivity. By changing the reaction solvent to ethanol, the reaction was transformed from a heterogeneous reaction to a homogeneous one. Therefore, the yield of 4-(α-bromoacetyl)-guaiacol-[α-^13^C] was increased from 81.08% to 94.84%. The optimal bromination reaction time was determined to be 1 h, with further extension of the reaction time having no significant effect on the product yield.

(2) The intermediate product, 4-(α-bromoacetyl)-guaiacol-[α-^13^C], could not be recrystallized using benzene and required purification via silica gel chromatography. Effective separation of the product was achieved using an eluent composed of a mixture of ethyl acetate/n-hexane (1/2 *v*/*v*).

(3) After the phenolic lignin model compound, i.e., guaiacyl glycerol-β-guaiacyl ether-[α-^13^C], was cooked using the kraft method in the presence of xylose, some benzyl ether linkages were formed between the α position of the lignin side and the xylose derivatives (uronic acid, etc.). These newly formed LCC structures were mainly found in ethyl acetate extractives.

## Figures and Tables

**Table 1:** Effect of reaction time on bromine reaction.

Reaction Solvent	Time (h)	Cuprous Bromide Recovery Rate	Product Yield
Ethyl alcohol	1	72.70%	94.84%
	2	83.28%	94.27%
	3	84.28%	93.01%
Trichloromethane/ethyl acetate (1/1, *v*/*v*)	1	92.04%	81.08%
	2	92.44%	81.37%
	3	92.36%	80.37%

**Table 2:** Assignments of signals in ^1^H-NMR spectrum of 4-(a-bromoacetyl)-guaiacol.

Signal	δ (ppm)	Assignments
1	7.59–7.61	proton on C_2_ and C_6_ in guaiacyl
2	6.85–7.57	aromatic protons
3	5.26	methylene
4	4.92	bromomethyl
5	3.82	methoxy group

**Table 3:** Assignments of signals in ^1^H-NMR spectrum of guaiacyl glycerol-β-guaiacyl ether.

Signal	δ (ppm)	Assignments
1	6.82–7.24	aromatic protons
2	4.94	methylene in α position
3	3.98–4.13	proton in β position
4	3.80	methoxy group
5	3.43	proton in γ position

**Table 4:** The ^13^C-NMR signal assignment of guaiacyl glycerol-β-guaiacyl ether-[α-^13^C].

Signal	δ (ppm)	Assignments
1	149.8–149.9	C_3_ in guaiacyl
2	148.2–148.5	C_3_/C_4_ in guaiacyl
3	145.6–147.1	C_3_/C_5_ in guaiacyl
4	133.1–133.4	C_1_ in guaiacyl
5	120.8–121.8	C_6_ in
6	119.1–119.6	C_5_ in guaiacyl
7	111.1–115.9	C_2_ in guaiacyl
8	83.8–84.6	C_β_ in β-O-4
9	70.3–71.7	C_α_ in β-O-4
10	60.2	C_γ_ in β-O-4
11	55.5–55.7	methoxy group

**Table 5:** The assignments of signals in ^13^C-NMR spectrum of ethyl acetate extractive.

Signal	δ (ppm)		Assignments
	A	B	
2	191.2	191.2	C=O in α position
4	147.8	147.8	C_4_ in etherified guaiacyl
6	133.0		C_1_ in etherified guaiacyl
7	130.9	130.9	C_1_ in etherified guaiacyl
9	121.0	121.0	C_6_ in etherified guaiacyl
10	119.4	119.3	C_5_ in etherified guaiacyl
11	115.6	115.7	C_5_ in etherified guaiacyl
12	112.5	112.4	C_2_ in etherifiedguaiacyl
13	109.7		C_2_ in guaiacyl
14	98.7		C_1_ in xylose
15	81.5		C_α_ (β-O-4) with ether bond to xylaose
16	79.3		C_β_ in guaiacyl
17	77.8		C_3_/C_4_ in xylose
18	76.5		C_3_ in xylose
19	75.6, 74.2		C_2_ in xylose
20	72.8		C_2_/C_3_ in xylose
21	71.6, 71.3	71.3, 70.9	C_α_ in β-O-4
22	66.0		C_5_ in xylose
23	55.8	55.9	methoxyl group

## Data Availability

The data presented in this study are available in the manuscript.
